# Interest–Ability Profiles: An Integrative Approach to Knowledge Acquisition

**DOI:** 10.3390/jintelligence10030043

**Published:** 2022-07-16

**Authors:** William E. Hyland, Kevin A. Hoff, James Rounds

**Affiliations:** 1 Department of Psychology, University of Illinois at Urbana-Champaign, Champaign, IL 61820, USA; jrounds@illinois.edu; 2 Department of Psychology, University of Houston, Houston, TX 77204, USA; khoff@uh.edu

**Keywords:** vocational interests, cognitive ability, knowledge acquisition, trait complexes, PPIK

## Abstract

Cognitive abilities and interests both play an important role in guiding knowledge acquisition, but most previous studies have examined them separately. The current study used a large and representative dataset to integrate interests and abilities using a person-centered approach that examines how distinct profiles of interests and abilities relate to individual strengths and weaknesses in knowledge. Two key findings emerged. First, eight interest–ability profiles were generated from Latent Profile Analysis (LPA), which replicated and extended the interrelations of interests and abilities found in previous studies using variable-centered approaches. Second, each profile’s strongest knowledge scores corresponded to their strongest abilities and interests, highlighting the importance of interest–ability profiles for guiding the development of knowledge. Importantly, in some domains, the lower ability profiles were actually more knowledgeable than higher ability profiles. Overall, these findings suggest that people learn best when given opportunities to acquire knowledge relevant to both their interests and abilities. We discuss how interest–ability profiles inform integrative theories of psychological development and present implications for education and career development.

## 1. Introduction

The influence of cognitive abilities and vocational interests pervades almost every stage of professional development, from high school course preferences (Lavrijsen et al. 2021; Wai et al. 2009) to university majors (Ackerman and Beier 2003) and university GPAs (Berry and Sackett 2009; Nye et al. 2012), as well as eventual career choices (Lubinski and Benbow 2006; Tracey and Hopkins 2001) and job performance (Nye et al. 2017; Schmidt and Hunter 2016; Van Iddekinge et al. 2011). The combined effect of interests and abilities on educational and work outcomes can be explained by theories of intellectual development (Ackerman 1996; Holland 1973), which posit that interests and abilities are mutually reinforcing in the development of domain-specific knowledge; that is, individuals are more likely to attempt to learn information they are interested in, and if they are able to learn that information successfully, their interest in it is likely to increase, leading to further attempts at learning. However, most studies on abilities and interests have used a “siloed” approach (Kanfer et al. 2010), in which these variables were investigated separately. Studies that have integrated interests and abilities have made important theoretical contributions to understanding how different types of interests relate to different abilities (Achter et al. 1999; Ackerman 1997; Johnson and Bouchard 2009), as well as their role in acquiring knowledge (Ackerman et al. 2001; Rolfhus and Ackerman 1999). Yet, the existing research has only examined knowledge acquisition using variable-centered approaches, which do not consider the within-person profiles of abilities and interests that influence it.

Examining abilities and interests at the within-person level is important because, in the real world, people often make career decisions based on their strongest and weakest interests and abilities. For example, two students with high levels of spatial ability are likely to pursue divergent career paths if they differ on “people vs. things” interests (Lubinski 2000, p. 424). This highlights the importance of assessing interests and abilities together to better understand career choices and outcomes (McCloy et al. 2020). Analyzing interests and abilities using a person-centered approach^1^ can also test the core assumptions of intellectual development theories. In particular, person-centered approaches are necessary to examine how distinct profiles of interests and abilities predict people’s relative strengths and weaknesses in knowledge areas.

The present study uses a large and representative dataset (Project Talent; Flanagan et al. 1962) to investigate within-person differences in cognitive abilities and vocational interests using an integrative, person-centered approach. Our study has two main aims. First, we examine the characteristics of distinct interest–ability profiles using latent profile analysis. Analyzing the profiles of interests and abilities informs integrative models of individual differences (Ackerman and Heggestad 1997; Austin and Hanisch 1990; Bernstein et al. 2019; Johnson and Bouchard 2009) by revealing how different combinations of interests and abilities co-occur at the within-person level. We also contribute new findings about how gender and socioeconomic status relate to interest–ability profiles. Our study’s second major aim is to evaluate the central tenet of intellectual development theories: that individuals are most knowledgeable in areas in which they are most interested and have the greatest ability (Ackerman 1996; Holland 1973; Schmidt 2014). We test this assumption by examining how different interest–ability profiles relate to knowledge scores in 14 areas. The Project Talent dataset is uniquely suited to these analyses because it is the largest available dataset that contains measures of vocational interests, cognitive abilities, and knowledge. Furthermore, no studies have been conducted on how interests and abilities jointly contribute to knowledge in this dataset. Overall, our study has important implications for understanding how interests and abilities work together to direct knowledge acquisition, which ultimately guides people’s educational trajectories and career trajectories.

### 1.1. Vocational Interests and Cognitive Abilities

Vocational interests are trait-like preferences for work activities, environments, or outcomes that motivate goal-oriented behaviors (Rounds and Su 2014). The most prominent framework for studying interests is the RIASEC model (Holland 1997), which organizes interests into six types: Realistic, Investigative, Artistic, Social, Enterprising, and Conventional. *Realistic* interests involve hands-on activities and manual labor. *Investigative* interests involve scientific inquiry and complex thinking. *Artistic* interests involve the fine arts, such as theater, music, and writing. *Social* interests involve working with and helping others. *Enterprising* interests involve persuasiveness and competitiveness. Finally, *Conventional* interests involve attention to details and records (Hanna et al. 2021, p. 3; Holland 1959).

The structure of cognitive ability was debated throughout the 20th century, but there is now a general consensus. Most modern models of ability include several narrow abilities organized hierarchically, with a general factor of intelligence, also referred to as “g” or “general mental ability” (GMA), at the top of the hierarchy (Carroll 1993). This “g” factor can be broadly defined as “a very general mental capability that, among other things, involves the ability to reason, plan, solve problems, think abstractly, comprehend complex ideas, learn quickly and learn from experience” (Gottfredson 1997, p. 13). The specific abilities that occupy the second stratum of the intelligence hierarchy are less agreed upon, with prevalent models specifying anywhere from 3 (Guttman 1957; Johnson and Bouchard 2005) to 8 (Schneider and McGrew 2012) abilities. Despite these differences, all major intelligence models contain at least three specific abilities, and these abilities are similar across all models. These abilities are verbal, quantitative, and spatial ability (Guttman 1957; Snow 1978). Verbal ability is measured by performance in linguistic tasks, such as reading comprehension. Quantitative ability is measured by performance in mathematical tasks, such as solving equations. Finally, spatial ability is measured by performance in geometric tasks, such as object rotation.

Some attempts have been made to examine interests and abilities jointly. Integrative models of individual differences, such as PPIK (Ackerman 1996) and Schmidt’s (2014) theoretical model, have established frameworks for how these traits relate to one another developmentally, leading to the acquisition of knowledge and skills. Other studies have analyzed interests and abilities in relation to outcomes such as school subject preferences (Lavrijsen et al. 2021), college major (Achter et al. 1999), occupational choice (Austin and Hanisch 1990), and professional eminence (Bernstein et al. 2019). Meta-analyses have also provided evidence for the correlations between these variables (Ackerman 1997; Pässler et al. 2015). However, none of these studies have investigated abilities and interests using a person-centered approach, which offers a key advantage: it can account for intrapersonal patterns of traits. Take for example the decision to pursue a STEM college major. Variable-centered approaches can estimate how important certain traits are for choosing a STEM major, but they cannot estimate the importance of distinct patterns of traits. We next review trait complex theories and explain how person-centered approaches can better inform understanding of knowledge acquisition.

### 1.2. Trait Complexes and Knowledge Acquisition

Trait complexes are clusters of individual differences from traditionally disparate domains (Ackerman and Heggestad 1997). When applied to cognitive abilities and vocational interests, trait complexes tend to form at least three clusters: the science/math complex, the intellectual/cultural complex, and the social complex (Ackerman et al. 2001, 2013). The science/math complex contains quantitative ability, spatial ability, Realistic interests, and Investigative interests. The intellectual/cultural complex contains verbal ability, Investigative interests, and Artistic interests. Finally, the social complex contains Social interests and Enterprising interests.

One of the primary purposes of trait complex theory is to account for individual differences in the acquisition of knowledge, which is requisite for the development of skills and expertise (Ackerman 1996; Ackerman and Heggestad 1997). To explain differences in knowledge acquisition, Ackerman proposed a model called PPIK, which stands for “intelligence-as-Process, Personality, Interests and intelligence-as-Knowledge” (Ackerman 1996). This model posits two stages of intellectual development. The first, often referred to as “intellectual investment”, is based on Cattell’s theory of intelligence, which posits two types of intelligence: “fluid” and “crystallized” (Cattell 1963). Fluid ability (“intelligence-as-Process”) represents a pure capacity for learning, whereas crystallized ability (“intelligence-as-Knowledge”) represents the knowledge gained by investing fluid ability. This stage of investment has been extended by the Openness-Fluid-Crystallized-Intelligence (OFCI) model, which specifies the mechanisms by which the personality trait of openness to experience influences the development of fluid and crystal abilities (Ziegler et al. 2012). Indeed, research on the OFCI model has found that openness interacts with fluid intelligence when predicting crystallized abilities such as math and reading competence (Lechner et al. 2019).

The second stage involves the further development of crystallized abilities and interests along mutually causal lines (Holland 1973). Specifically, interests determine an individual’s motivation for attempting a particular task, and abilities determine the individual’s probability of success at that task. After several successful attempts at a task, interest in the task increases, as well as task-relevant knowledge. Conversely, after several unsuccessful attempts at a task, interest in the task may decrease, leading to fewer future attempts and knowledge gains.

PPIK specifies the interest traits that are relevant to the development of specific kinds of knowledge. According to PPIK, Realistic interests lead to knowledge of physical science and mathematics, Investigative interests lead to knowledge of physical science and social science, and Artistic interests lead to knowledge of literature and the arts (Ackerman 1996). Although not directly included in the model, Social and Enterprising interests have also been shown to relate to certain kinds of knowledge (Reeve and Hakel 2000). Overall, PPIK provides a unifying framework that explains how trait complexes of interests and abilities lead to the development of different kinds of knowledge.

### 1.3. Person-Centered Approach to Trait Complexes

Trait complex theory is a variable-centered model of individual differences because it focuses on how levels of specific traits are related to each other. In contrast, a person-centered model of individual differences is concerned with profile shape, or how the levels of a person’s traits relate to each other (Hofmans et al. 2020; Woo et al. 2018). Evaluating the profile shape of abilities and interests is critical because it provides information that is unavailable when only comparing traits between persons (Lubinski 2020).

Profile shape is a key component of vocational interest assessment. Individuals are more likely to pursue careers in which they are most interested (relative to other careers), so within-person interest profiles are crucial for predicting career choices (Hanna and Rounds 2020). Interest profiles are also important predictors of STEM major choice (Perera and McIlveen 2018) and mathematical literacy (Warwas et al. 2009).

In the area of cognitive abilities, different profile shapes also result in disparate outcomes. Specifically, individuals with dominant verbal abilities, or “verbal tilt”, are more likely to have aesthetic values (Bernstein et al. 2019) and literary-linguistic interests (Humphreys et al. 1993), prefer classes in the humanities and social sciences, and enter occupations in these areas (Wai et al. 2009). Individuals with a spatial or mathematical tilt, on the other hand, tend to have theoretical values and physical science interests, prefer classes in math and science, and enter occupations in these fields.

Overall, studies on the profile shape of interests and abilities show that person-centered approaches to individual differences reveal important information not directly available from variable-centered approaches. To date, however, no studies that we are aware of have investigated profiles of interests and abilities or how such profiles relate to applied outcomes. We address this limitation in the current study to inform understanding about how distinct combinations of interests and abilities predict the acquisition of knowledge in different areas.

### 1.4. The Present Study

The aim of this study is to investigate profiles of interests and abilities and the extent to which these profiles are associated with strengths and weaknesses in knowledge. To accomplish this, we employ latent profile analysis (LPA), a person-centered method that groups individuals with similar levels of traits into different profiles rather than grouping items or variables based on their similarity (Hofmans et al. 2020). By producing such profiles, LPA allows us to investigate how different patterns of interests and abilities lead to different kinds of knowledge. Ultimately, the goal is not merely to predict which profiles will be the most knowledgeable but to compare profiles to each other to understand their relative strengths and weaknesses in knowledge.

In general, we expect that the profiles produced by LPA will be consistent with relevant theories, including intellectual development (Ackerman 1996; Holland 1973), trait complexes (Ackerman and Heggestad 1997), and Holland’s vocational theory (1997). Intellectual development theories state that individuals’ interests and abilities are mutually reinforcing such that people tend to like things they are good at and be good at things they like. Thus, we would expect a profile’s relatively high ability to line up with its relatively high interest. Trait complexes and Holland’s vocational theory elaborate upon the expected interest–ability relations, which we utilize to develop hypotheses about the expected interest–ability profiles. For example, these theories posit relations between verbal ability and Artistic interests, so we would expect a profile with a relatively high verbal ability to have relatively high Artistic interests.

Trait complex studies have consistently produced a science/math complex, which consists of Realistic interests, Investigative interests, quantitative ability, and spatial ability (Ackerman et al. 2001, 2013). This grouping of traits is also supported by Holland’s vocational theory, which posits that individuals with Investigative interests tend to have scientific and mathematical abilities (1997). Furthermore, research on ability profiles has demonstrated relations between spatial tilt and physical science interests, which correspond to Realistic and Investigative interests (Humphreys et al. 1993). Extending this to interest–ability profiles, we propose the following:

**Hypothesis** **1a.** *There will be one or more profiles characterized by dominant spatial ability, quantitative ability, Realistic interests, and Investigative interests.*

Trait complex studies have also consistently produced an intellectual/cultural complex, which consists of verbal ability, Investigative interests, and Artistic interests (Ackerman et al. 2001, 2013). This grouping of traits is supported by Holland’s (1997) theory, which posits that individuals with Artistic interests tend to have some ability in writing and speaking. Furthermore, research on profiles of abilities has demonstrated substantial relations between verbal tilt and literary-linguistic interests, which correspond to Artistic interests (Humphreys et al. 1993). Thus, we propose the following:

**Hypothesis** **1b.** *There will be one or more profiles that are characterized by dominant verbal ability, Artistic interests, and Investigative interests.*

Lastly, the social complex appears in most trait complex studies and is comprised of Social and Enterprising interests. This grouping of traits is somewhat supported by Holland’s (1997) vocational theory, which posits that individuals with Social interests tend to lack mechanical and scientific ability. The lack of an ability associated with Social and Enterprising interests can also be understood in terms of intellectual development theories (Ackerman 1996; Holland 1973), where individuals with low academic ability could compensate for this deficit by developing social skills aimed at helping others (Social interests) or persuading others (Enterprising interests). Thus, we propose the following:

**Hypothesis** **1c.** *There will be one or more profiles which are characterized by average-to-below-average levels of ability and dominant Social and Enterprising interests.*

To better understand the context in which profiles of interests and abilities develop, we also examine them in relation to gender and socioeconomic status (SES). Children are differentially exposed to learning opportunities and rewards for engaging in these opportunities depending on their gender and SES (Lent et al. 1994), so it is likely that profile membership will be influenced by these two variables. Although gender equality in American society has greatly improved since 1960s, when our data were obtained, gender differences in interests and abilities have persisted across cohorts (Halpern 2012; Hansen 1988; Hedges and Nowell 1995; Su et al. 2009).

The largest disparities between genders is in Realistic interests (d = 0.84), favoring men, and Social interests (d = −0.68), favoring women (Su et al. 2009). Smaller differences have been observed for abilities, with women scoring slightly higher than men on verbal ability and slightly lower on spatial ability (Linn and Petersen 1985; Maeda and Yoon 2013). Abilities are also related to SES (ρ = 0.21–0.56) (Strenze 2007). However, since little research has examined how gender and SES relate to within-person profiles of interests and abilities, we pose the following questions:

**Research** **Question** **1.** *How do (***RQ1a***) gender and (***RQ1b***) SES influence the probability of an individual belonging to a particular interest–ability profile?*

Our second set of analyses focuses on how trait profiles predict 14 knowledge areas in 4 broad categories of knowledge: humanities (art, the Bible, literature, music, and theater), civics (business, law, and social studies), science (biology, math, and physics) and mechanical (aeronautics, electronics, and mechanical). These domains are similar to the knowledge scales developed in Rolfhus and Ackerman’s (1999) study on adult intelligence, which were used in subsequent trait complex studies. Assuming our interest–ability profiles mirror the trait complexes as outlined by the first set of hypotheses, we also expect they will be related to domain knowledge in a similar way.

The math/science complex has been shown to correlate positively with all knowledge areas, but it relates most strongly to science knowledge (Ackerman et al. 2001). Research on interests and knowledge also demonstrates relations between Realistic and Investigative interests with physical science knowledge (Reeve and Hakel 2000; Rusche and Ziegler 2022). In addition, individuals with high spatial or mathematical ability are more likely to select majors in the physical sciences, suggesting a propensity for this subject matter (Wai et al. 2009). Therefore, we propose the following:

**Hypothesis** **2a.** *Profiles characterized by dominant spatial ability, quantitative ability, Realistic interests, and Investigative interests will have higher levels of physical science knowledge compared with other knowledge domains.*

The intellectual/cultural complex has also been shown to correlate positively with general levels of knowledge, but it relates most strongly to humanities knowledge (Ackerman et al. 2001). Research has also demonstrated relations between Investigative and Artistic interests with humanities knowledge (Reeve and Hakel 2000). Furthermore, individuals with a relatively high verbal ability are more likely to choose majors in the humanities and social sciences, indicating their preference for this subject matter (Wai et al. 2009). Thus, we propose the following:

**Hypothesis** **2b.** *Profiles characterized by dominant verbal ability, Artistic interests, and Investigative interests will have higher levels of humanities knowledge compared with other knowledge domains.*

The social complex correlates negatively with most knowledge areas, but its weakest negative relation is with civics knowledge (Ackerman et al. 2001). Research on interests and knowledge also demonstrates negative or insignificant relations between Social interests and all areas of knowledge, except social studies (Reeve and Hakel 2000). Extending this to within-person profiles, we propose the following:

**Hypothesis** **2c.** *Profiles characterized by dominant Social interests will have higher levels of civics knowledge compared with other knowledge domains.*

## 2. Method

### 2.1. Participants

The participants were drawn from the Project TALENT study (Flanagan et al. 1962), which included a stratified sample of 5% of American high school students from grades 9–12. There were 375,015 participants in the original sample, with 188,174 (49.9%) males and 188,841 (50.1%) females. Among the participants who indicated their race or ethnicity, 147,471 (94.40%) were White or Caucasian, 6549 (4.19%) were Black or African American, 1022 (0.65%) were Asian American, 625 (0.40%) were Latino or Hispanic, 415 (0.27%) were Native American, and 88 (0.06%) responded “other”. Although these proportions depart from the actual racial composition of the U.S. in 1960, this is likely due to the low response rate for the race or ethnicity item, which was less than half. The participants were subjected to an extensive battery of surveys and tests, ranging from demographic characteristics to interests, aspirations, and cognitive abilities. Although follow-up surveys were conducted 1, 5, 11, and 50 years after the original testing (Damian et al. 2019; Wise et al. 1979), the current study only focuses on the data from the initial survey.

### 2.2. Measures

#### 2.2.1. Cognitive Abilities

Integrative and within-person studies on abilities have primarily focused on verbal, quantitative, and spatial abilities (Ackerman 1997; Austin and Hanisch 1990; Lubinski 2010; Shea et al. 2001). Since the current study is a constructive replication, we selected a model that represents these three abilities called the radex model (Guttman 1957; Snow 1978). This model has been applied to Project Talent ability scales in previous studies (Humphreys et al. 1993; Wai et al. 2009), and we followed their example in constructing the scale composites.

The *verbal composite* consisted of three tests: the vocabulary test had 30 items measuring general knowledge of words, the English test had 113 items assessing spelling, grammar, and language expression, and the reading comprehension test had 48 items measuring comprehension of texts. The *quantitative composite* consisted of four tests: mathematics had 23 items measuring knowledge of math definitions and notation, arithmetic reasoning had 16 items that measured the ability to solve basic arithmetic problems, introductory mathematics had 24 items measuring all forms of math knowledge taught through the 9th grade, and advanced mathematics had 14 items covering topics taught in grades 10–12, such as algebra, geometry, probability, logic, and basic calculus. The *spatial composite* also consisted of four tests: the two-dimensional spatial visualization test had 24 items measuring the ability to visualize two-dimensional figures that were rotated on a plane, three-dimensional spatial visualization had 16 items measuring the ability to visualize two-dimensional figures after they were folded into three-dimensional figures, mechanical reasoning had 20 items measuring the ability to deduce relationships between gears, pulleys, and springs, as well as knowledge of basic physical forces, such as gravity, and abstract reasoning had 15 items that involved finding logical relationships in sophisticated figure patterns.

The intercorrelations among these composites were 0.62 for math-spatial, 0.59 for verbal-spatial, and 0.70 for math-verbal. Table S5 in the Supplementary Materials presents the names of the tests, the number of items in them, and their reliabilities.

#### 2.2.2. Interests

Project Talent’s interest inventory contains 205 items rated on a scale from 1 (“I would dislike this very much”) to 5 (“I would like this very much”). The items consist of different occupations, such as “construction worker” or “biologist”. These interest items were originally organized into 17 interest factors that resembled modern basic interest scales (Liao et al. 2008). However, as this study is a constructive replication, it was necessary to represent interests with the RIASEC model (Holland 1959, 1997). We derived the RIASEC scales by matching items to Holland’s (1965) Vocational Preference Inventory, resulting in 9-item scales for each interest. The internal consistencies of these scales ranged from Cronbach’s α = 0.83 (conventional) to α = 0.88 (realistic). Table S1 reports the items that comprise each scale.

#### 2.2.3. Domain Knowledge

In the original Project Talent assessment, the participants completed 38 domain-specific information tests designed to measure their knowledge of academic (e.g., physical sciences) and nonacademic subjects (e.g., the outdoors, hunting, and fishing). The number of items in each test ranged from 3 (colors) to 24 (literature). For the current study, we included all tests that were academic in nature and relevant to intellectual development (i.e., excluding tests measuring attitudes, non-specific information, or obscure domains). This resulted in 14 knowledge scales covering 4 broad disciplines: the humanities, civics, science, and mechanical. These knowledge scales were similar to those used in prior studies on trait complexes (Ackerman 2000; Rolfhus and Ackerman 1999). Table S6 in the Supplementary Materials presents the names of the tests, the number of items in them, and their reliabilities. Additional details on the development and validity of these knowledge tests are available in Flanagan et al. (1962) or the Project Talent data bank handbook (Wise et al. 1979).

### 2.3. Analytical Strategy

The data were analyzed in a three-step process. First, we used factor analysis to investigate the structure of the study’s variables, which is recommended prior to conducting latent profile analysis (Spurk et al. 2020). Second, we constructed profiles of abilities and interests using latent profile analysis (LPA). Third, we used the identified profiles to predict domain knowledge using a series of regression models.

#### 2.3.1. Exploratory Factor Analysis

Factor analysis is recommended as a first step prior to LPA in order to confirm that the study’s variables conform to the theoretically expected higher-order structure (Spurk et al. 2020). Since one of the main purposes of this paper is to demonstrate that interest–ability relations are better represented at the within-person level (i.e., with a profile) with respect to knowledge, we did not expect a factor analysis of interests, abilities, and knowledge to result in a clear, three-factor structure. Indeed, following trait complex research, we consider the relations between these domains to indicate an important overlap between interests, abilities, and knowledge. Thus, we included the results of an exploratory factor analysis of the study’s interest, ability, and knowledge variables. Table S3 in the Supplementary Materials presents the factor loadings, and Figures S1 and S2 present the scree plot method and Horn’s parallel analysis method for determining the number of factors.

#### 2.3.2. Latent Profile Analysis

LPA is a categorical, latent variable approach that uses a set of variables to identify subgroups within a population (Spurk et al. 2020). LPA has become an increasingly valuable tool for assessing combinations of individual differences, both in vocational (Howard et al. 2016; Meyer et al. 2015) and personality (Gerlach et al. 2018; Merz and Roesch 2011) research. LPA has three main advantages over simpler clustering techniques, such as centroid-based clustering (Hofmans et al. 2020). First, LPA uses “soft clustering”, which means that it calculates the probability that individuals belong to each class rather than directly assigning them to one class. This allows for a more nuanced view of class membership and helps avoid strict typologies. Second, LPA considers the distributions of each variable in the model, allowing for model selection and goodness of fit indices. Third, LPA can be conducted with covariates, allowing for interpretation of profiles within a nomological network.

LPA was performed in Mplus version 8.6 (Muthén and Muthén 2017) using a robust maximum likelihood estimator. To compare different models, we considered three statistical fit indices: the Akaike information criterion (AIC), Bayesian information criterion (BIC), and sample-adjusted Bayesian information criterion (SABIC). We mainly relied on the BIC and SABIC, since these are considered the most accurate (Morgan 2015; Nylund et al. 2007) and perform well in large samples (Spurk et al. 2020; Tofighi and Enders 2008). Furthermore, we used the bootstrap likelihood ratio test (BLRT) and the adjusted Lo–Mendell–Rubin test (LMR) to evaluate the relative fit of a *k* profile model versus a *k* − 1 profile model, where a nonsignificant *p*-value indicates the superior fit of the *k* − 1 profile model. Lastly, we examined the entropy values for each model solution, with higher values indicating higher classification accuracy. The threshold for acceptable fit is generally considered to be 0.8 or higher (Tein et al. 2013).

We also included gender and SES as covariates in our analysis using Mplus’s DCAT function (Asparouhov and Muthén 2014; Lanza et al. 2013). This function performs equality χ^2^ tests of the covariate’s means and probabilities for each class without including it in the LPA model, leaving the initial profile solution unaffected. Comparing class-specific means and probabilities allowed us to parse the effects of gender and SES on membership for each interest–ability profile, which we reported as probabilities and means.

#### 2.3.3. Profiles as Predictors of Domain Knowledge

Our second aim was to predict within-person differences in domain knowledge using interest–ability profiles. To this end, we used each interest–ability profile to predict each knowledge scale. Specifically, we used linear regression in Mplus to regress the knowledge scales on class membership probabilities for each profile, producing standardized beta coefficients that represent the strength of the relationship between each profile and each knowledge scale. Then, we plotted these coefficients to create a knowledge profile for each interest–ability profile, allowing for side-by-side comparison of the interest–ability profiles and knowledge profiles.

### 2.4. Data, Materials, and Code

The Project Talent dataset is protected by a Restricted Use of Data Agreement, which prevents us from making it publicly available. However, we provided the code used to analyze the data, which is available at our Open Science Framework page. The data were analyzed using MPlus version 8.6 (Muthén and Muthén 2017) and visualized using R version 4.1.1 (R Core Team 2021) and the package ggplot version 3.3.5 (Wickham 2016). Sample sizes of at least 500 participants are recommended for adequate power, which we exceed by a wide margin (Spurk et al. 2020). This study’s design and analyses were not preregistered.

## 3. Results

Table 1 reports the descriptive statistics and correlations between the interests, abilities, gender, and SES. For correlations with knowledge domains, see Table S2 in the Supplementary Materials. Table 2 reports the fit indices for the LPA models, ranging from 2 to 10 profiles.

To choose the optimal profile solution, we followed the best practice procedure recommended by Spurk et al.: (1) check for error messages, out-of-bounds parameters, and theoretical plausibility, (2) compare the remaining models with respect to the relative fit information criteria (e.g., AIC, BIC, and SABIC), (3) evaluate the reliability of the class assignment (e.g., entropy), and (4) compare the likelihood ratio tests (2020). Since our analysis did not produce any error messages or out-of-bounds parameters, we began by evaluating the theoretical plausibility of each profile solution. With the exception of the two-, three-, and four-profile models, which contained profiles with uniformly high or low levels of traits, all of the profile solutions were theoretically coherent. Next, we turned to the relative fit information criteria, examining each profile solution’s AIC, BIC, and SABIC. Information criteria indicate the optimal solution when the values reach a minimum and then start to ascend. Since none of the values reached a minimum, we were unable to use these criteria to select a particular profile solution. We next evaluated the profile solutions’ reliability of assigning individuals to profiles, which was measured by entropy scores. The two-profile solution had the highest entropy score, but it was theoretically incoherent, so we examined the model with the next highest score: the eight-profile solution. The 8-profile solution had an entropy score of 0.791, which is close to the optimum value of 0.80 (Clark and Muthén 2009). The reliability of profile assignment in the 8-profile solution ranged from 0.779 to 0.908, indicating that individuals were assigned to the same profile 78–91% of the time, depending on which profile they belonged to. Lastly, we compared the likelihood ratio tests (i.e., BLRT and Adj. LMR), which indicated whether a k-profile solution was less parsimonious than a k-1 profile solution. According to the adjusted LMR values, the three- and four-profile solutions were more parsimonious than the four- and five-profile solutions, respectively. Thus, statistical fit indices pointed to three potential profile solutions: three profiles, four profiles, or eight profiles. Considering these solutions holistically, we decided that the eight-profile solution was superior to the three- and four-profile solutions. Although the parsimony of the three- and four-profile solutions was attractive, these solutions were also the least reliable of all the profile solutions (i.e., had the lowest entropy scores) and were the least theoretically coherent (i.e., tended to contain profiles with uniformly high or low levels of traits). Therefore, we selected the eight-profile solution for its statistical fit and theoretical coherence. Membership in the 8 profiles ranged from 29,119 (9.1% of the sample) to 63,580 (19.9% of the sample). Table S4 in the Supplementary Materials displays the average latent class assignment probabilities for each profile.

### 3.1. Interpretation of the Interest–Ability Profiles

**Figure 1:**
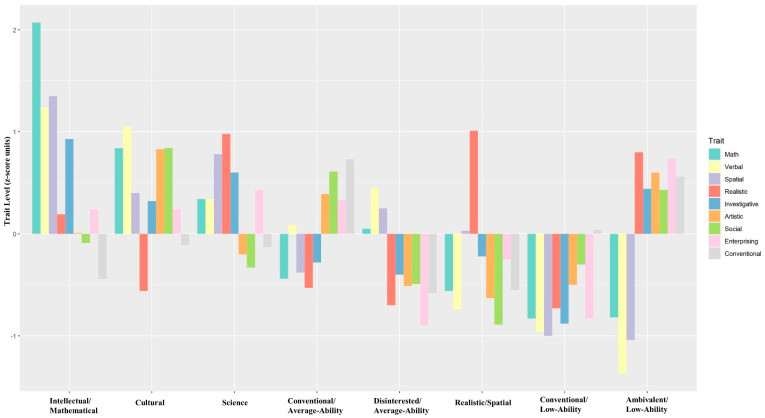
*Profiles resulting from latent profile analysis. Note*: The proportion of participants in each profile is as follows: intellectual/cultural = 9.76%, cultural = 11.29%, science = 13.55%, conventional/average-ability = 19.94%, disinterested/average-ability = 10.00%, realistic/spatial = 12.11%, conventional/low-ability = 14.21%, and ambivalent/low-ability = 9.13%. Standard errors for profile trait means were all less than 0.05 z-scores.

Our first set of hypotheses predicted that the interest–ability profiles would be similar to the trait complexes identified from the intercorrelations among interests and abilities. Figure 1 presents graphical representations of the eight profiles. Overall, three of the profiles aligned well with the hypothesized science/math, intellectual/cultural, and social trait complexes. This provided partial support for the first set of hypotheses. However, none of the profiles matched these complexes exactly, and some were entirely novel. We next review each profile in detail.

Hypothesis 1a predicted a profile analogous to the science/math complex, characterized by high spatial ability, quantitative ability, Realistic interests, and Investigative interests. One of the profiles met three of the four criteria: spatial ability, Realistic interests, and Investigative interests. Since quantitative abilities were not especially pronounced in this profile, we removed “math” from the title and named it the “Science” profile.

Hypothesis 1b predicted a profile similar to the intellectual/cultural complex, characterized by dominant verbal ability, Investigative interests, and Artistic interests. The profile closest to this description had dominant verbal abilities and Artistic interests but had much higher Social interests than Investigative interests. Thus, we removed “Intellectual” from the title and named it the “Cultural” profile. Interestingly, another profile emerged with characteristics of both the science/math complex and the intellectual/cultural complexes. This profile had the highest overall abilities, and we labeled it “Intellectual/Mathematical” due to its dominant mathematical ability and Investigative interests. 

Hypothesis 1c predicted a social profile characterized by average-to-below-average levels of abilities and dominant Social interests. This prediction was not supported. The results did, however, yield two profiles matching a trait complex not expected to replicate: the clerical/conventional complex (Ackerman and Heggestad 1997). These profiles both featured dominant Conventional interests and average-to-below-average abilities, which is consistent with previous research. Accordingly, we named these profiles “Conventional/Low-Ability” and “Conventional/Average-Ability”.

Three additional profiles appeared that were not hypothesized. One of these showed a distinct pattern of abilities and interests with no precedent in trait complex research. In fact, it had the greatest disparity between the highest interest (Realistic) and lowest interest (Social) of any profile, with nearly two standard deviations between them. The disparity between its highest ability (spatial) and lowest ability (verbal) was also the second highest of any profile. In light of these disparities, we named the profile after its dominant traits: “Realistic/Spatial”.

The other two unexpected profiles exhibited uniform levels of interest: one above average and the other below average. Such profiles are often referred to as “neutral” (Slot et al. 2020), “flat” (Leuty et al. 2015), “disinterested” (McLarnon et al. 2015), or “ambivalent” (Perera and McIlveen 2018). We added ability descriptors to these labels and named the profiles “Disinterested/Average-Ability” and “Ambivalent/Low-Ability”.

Research Questions 1a and 1b investigated the effects of gender and SES on profile membership. Table 3 and Table 4 present these conditional probabilities, expressed as proportions and means. The results indicated that profile membership was strongly influenced by gender such that 7 out of 8 profiles were 95% comprised of a single gender. The effect of SES on profile membership was less pronounced, although the higher-ability profiles tended to have higher levels of SES than the lower-ability profiles. Overall, these results suggest that the development of interests and abilities is influenced by social context, especially one’s gender.

### 3.2. Profiles and Domain Knowledge

**Figure 2:**
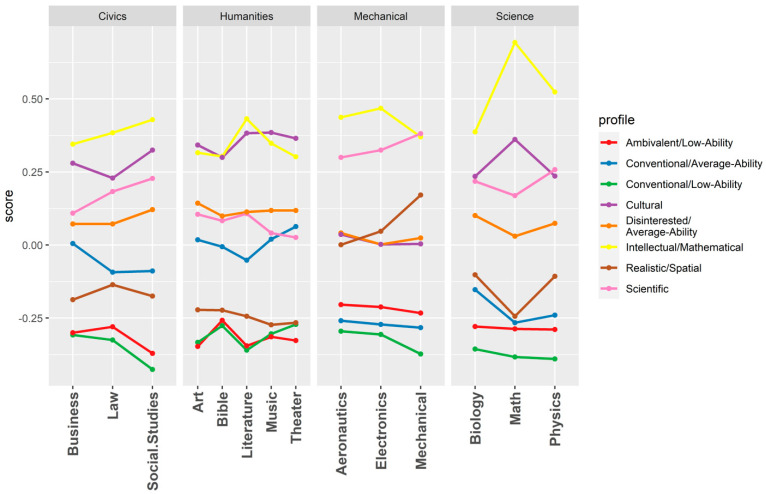
*Trait profile knowledge scores by domain. Note:* Scores on the y-axis are standardized beta coefficients. These scores are calculated by regressing each knowledge domain on profile membership scores for each profile. Standard errors ranged from 0.001 to 0.002.

We next used the profile membership scores to predict the knowledge test scores. In this way, we were able to construct a knowledge profile for each interest–ability profile, demonstrating the profile’s relative strengths and weaknesses. Figure 2 displays these results graphically, and Table 5 contains the beta coefficients from the regression analyses.

Hypothesis 2a predicted that profiles characterized by dominant spatial ability, quantitative ability, Realistic interests, and Investigative interests would be more knowledgeable about the physical sciences than other areas. This hypothesis was supported by the intellectual/mathematical profile, which scored the highest in physical science knowledge, and partially supported by the science profile, which scored second-highest in physical science after mechanical. Both the intellectual/mathematical and science profiles had similar weaknesses in knowledge, scoring lowest in the humanities and civics.

Hypothesis 2b predicted that profiles characterized by dominant verbal ability, Artistic interests, and Investigative interests would be more knowledgeable about the humanities than other areas. This hypothesis was supported by the cultural profile, which had the highest score of any profile in humanities knowledge. In contrast, the cultural profile scored relatively low in mechanical knowledge, ranking fourth relative to the other profiles.

Hypothesis 2c predicted that profiles characterized by dominant social interests would be more knowledgeable about civics than other areas. Since a profile with dominant Social interests was not produced by the analysis, this hypothesis was not supported. However, two profiles emerged that resembled a trait complex we did not expect to find: the clerical/conventional complex (Ackerman and Heggestad 1997). Both profiles scored in the bottom half of the interest–ability profiles for overall knowledge. The conventional/average-ability profile exhibited moderate variability in knowledge, scoring somewhat higher in the humanities and civics than in mechanical and physical science. In contrast, the conventional/low-ability profile exhibited little variability across the knowledge areas.

The remaining three interest–ability profiles also differed in their knowledge strengths and weaknesses. The realistic/spatial profile exhibited some of the largest knowledge disparities of any profile, scoring much higher in mechanical than any other area. The disinterested/average-ability profile had a relatively flat knowledge profile, performing slightly better in the humanities and civics than in mechanical and physical science. The ambivalent/low-ability profile also had a flat knowledge profile but performed slightly better in mechanical and physical science than in the humanities and civics. Overall, these results indicate that the knowledge scores substantially differed across the interest–ability profiles.

## 4. Discussion

The current study identified eight major interest–ability profiles and investigated the extent to which these profiles explained knowledge scores in a wide range of academic areas. Our integration of interests and abilities is notable because only a few studies have jointly analyzed interests and abilities (Achter et al. 1999; Ackerman and Heggestad 1997; Johnson and Bouchard 2009), and no studies that we are aware of have used a person-centered approach to investigate interests and abilities in relation to knowledge. Our findings contribute to integrative models of individual differences and extend previous studies on trait complexes using variable-centered approaches (Ackerman et al. 2001, 2013). Overall, two findings stand out. First, as predicted by intellectual development theories (Ackerman 1996; Holland 1973), the profiles with relatively flat levels of interests and abilities showed little variation in knowledge scores, whereas the profiles with more differentiated interests and abilities showed large disparities in knowledge. Second, although there was a trend for higher-ability profiles to have generally higher levels of knowledge, lower-ability profiles were often more knowledgeable than higher-ability profiles in the areas that matched their specific abilities and interests. In other words, the interest–ability profiles that aligned with specific knowledge areas routinely outperformed interest–ability profiles with less alignment, even if the other profiles had higher overall levels of ability. We next review these findings in detail while discussing the theoretical and applied implications.

### 4.1. Interest–Ability Profiles Replicate and Extend Trait Complex Research

One of the main findings of the current study is that many interest–ability profiles resemble the trait complexes identified using variable-centered analytic approaches. Specifically, five of the eight interest–ability profiles produced by our analysis (mathematical/intellectual, cultural, scientific, conventional/average-ability, and conventional/low-ability) showed patterns of traits consistent with prior studies on trait complexes and cognitive tilt (Ackerman et al. 2001; Humphreys et al. 1993). A growing body of evidence indicates that interests and abilities develop in tandem, a proposition that is in line with developmental theories (Gottfredson 1981, 2005; Lent et al. 1994) and meta-analyses of interests and abilities (Ackerman and Heggestad 1997; Pässler et al. 2015). Our results demonstrate that patterns of these interests and abilities can be operationalized as intrapersonal profiles, providing a useful supplement to variable-centered integrative approaches (Ackerman 1997). The distinctive patterns of interests and abilities captured by profiles highlight how these variables jointly influence career behavior in the real world, such as by guiding the decision to pursue a career in the physical sciences, social sciences, humanities, or skilled trades.

Our findings also extend integrative research by identifying new profiles of interests and abilities. These new profiles were likely uncovered because of our latent profile methodology, which allowed us to represent all interests and abilities in each cluster. The sample may have also played a role (Project Talent; Flanagan et al. 1962), given that it is much larger and more representative than the college student samples used in previous trait complex studies (Ackerman et al. 2001, 2013; Bernstein et al. 2019). Notably, two of the newly identified profiles were undifferentiated profiles: disinterested/average-ability and ambivalent/low-ability. Together, these profiles accounted for about 20% of the sample, which indicates that many high school students struggle with career indecision (Tracey and Darcy 2002). Similar profiles have been found in studies using only vocational interests (McLarnon et al. 2015; Perera and McIlveen 2018; Slot et al. 2020). These findings stress the importance of career guidance, which can help indecisive students develop stronger vocational identities. Vocational identities not only help students make career choices earlier (Savickas 1993), but they are also associated with better mental health (De Goede et al. 1999; Lopez 1989).

Another important finding was that gender and socioeconomic status (SES) influenced the likelihood of being a member of a particular profile. These results are in line with developmental models, which outline how gender and SES influence educational opportunities and experiences, leading to different interests and abilities (Gottfredson 1981, 2005). Since interests and abilities strongly predict career decisions (Achter et al. 1999; Austin and Hanisch 1990; Tracey and Hopkins 2001), interest–ability profiles may provide a target for interventions aimed at exposing youth to a wider range of career options. For example, students in the cultural profile, which is predominantly female and characterized by low Realistic interests, or the scientific profile, which is predominantly male and characterized by low Social interests, could be encouraged to explore career paths outside of stereotypical gender roles. In a similar way, career education and applied programs can help students from low-SES backgrounds develop stronger Investigative interests, which were disproportionately found in higher-SES profiles (Schneider and Young 2019).

### 4.2. Interest–Ability Profiles Guide Knowledge Acquisition

Our second set of results revealed that the interest–ability profiles differed substantially in knowledge scores, and these differences corresponded to each profile’s dominant interests and abilities. For example, the intellectual/mathematical profile, which had dominant math ability and Investigative interests, was most knowledgeable about math and physics and least knowledgeable in the humanities. On the other hand, the cultural profile, which had dominant verbal ability and Artistic and Social interests, was most knowledgeable about the humanities and less knowledgeable about science and mechanics. This pattern of findings offers a unique contribution that complements variable-centered studies on interests, abilities, and knowledge (Achter et al. 1999; Ackerman et al. 2001; Bernstein et al. 2019). Specifically, the findings highlight the importance of assessing individuals’ relative levels of interests and abilities. Someone may be interested in STEM and have high mathematical and spatial abilities, but if he or she has even higher verbal ability and more interest in the humanities, he or she is more likely to develop knowledge in that area (Lubinski 2020).

Within-person differences in knowledge also have important implications for understanding between-person differences in knowledge. Notably, lower-ability profiles were often more knowledgeable than higher-ability profiles in the areas that aligned with their specific abilities and interests. For example, the cultural profile, despite having lower overall levels of ability, was slightly more knowledgeable in the humanities (average β = .36) than the intellectual/mathematical profile (average β = .34). Overall, the profiles performed well in areas of high interest across the board, underscoring the critical role of interests in influencing not only the kind of information that is pursued but how persistently and vigorously that information is pursued (Su 2020). These findings provide one explanation for why measures of knowledge achievement, such as AP test scores and GRE subject test scores, have incremental validity over aptitude tests in predicting grades (Ackerman et al. 2013; Kuncel et al. 2001): they capture both interest and ability. A similar explanation can be applied to occupational outcomes. For example, interests have incremental validity over abilities in predicting job performance (Van Iddekinge et al. 2011) because they influence the direction, persistence, and vigor of individuals’ knowledge development (Su 2020).

Finally, another noteworthy finding was that the undifferentiated profiles had substantially less variability in knowledge than the differentiated profiles. This finding is consistent with the core logic of intellectual development theories: individuals with uniformly high interests and uniformly low interests are just as likely to pursue one area of knowledge as any other. This also raises an important issue: the lack of strengths in knowledge can put undifferentiated profiles at a disadvantage in educational and occupational contexts, since most individuals pursue areas of relative strength. Thus, it is critical for schools and guidance counselors to help disinterested and ambivalent students narrow down potential career paths so they can better compete with individuals who have already begun to develop expertise (Hoff et al. 2021).

### 4.3. Strengths, Limitations, and Future Directions

The current study had several methodological strengths. Using Project Talent’s large, diverse sample allowed us to generate interest–ability profiles that represent individuals from a variety of demographics and socioeconomic backgrounds. We also demonstrated the important role of gender and SES in predicting profile membership. Our focus on domain knowledge in high school is notable because this has not been investigated in previous integrative studies. High school is an especially important juncture in the process of knowledge acquisition, as students often start to make decisions about their future careers during this time. Another strength of the current study is the breadth of Project Talent’s domain knowledge scales. This wide coverage can help guide diverse students toward well-fitting career paths. For example, the inclusion of mechanical tests such as aeronautics and electronics allowed us to identify an area of strength for an otherwise academically disinclined profile: realistic/spatial.

There were also several limitations. First, the data collection for this study occurred in 1960, a different historical period in the United States. This difference is especially salient in relation to gender and racial inequality in the workforce (del Río and Alonso-Villar 2015). Second, we were unable to investigate the relations between interest–ability profiles and personality in the current study. Although personality is usually part of trait complex research (Ackerman and Heggestad 1997), researchers have had difficulty adequately adapting the Project Talent personality scales to the five-factor model (Damian et al. 2019).

Our study also raises several directions for future trait profile research. First, future research should investigate whether these interest–ability profiles can be replicated in other samples, especially more recent ones. Interest–ability profiles can also be used to analyze occupational outcomes such as career choices and expertise. Related to this, there is great potential to inform the understanding of person–environment fit by using interest–ability profiles to investigate fit in relation to outcomes such as job performance and satisfaction. Lastly, one of the primary goals of trait complex research is to integrate individual differences. Thus, examining intrapersonal profiles in relation to other traits such as personality, self-concept, motivation, or work values could help establish a more comprehensive integrative framework of individual differences.

## 5. Conclusions

The current study used latent profile analysis to construct profiles of cognitive abilities and vocational interests. A wide range of knowledge domains was then regressed on these interest–ability profiles, resulting in distinctive patterns of knowledge for each profile. Overall, our findings show that the interest–ability profiles resemble the trait complexes found in Ackerman et al.’s research, and we also discovered novel interest–ability profiles that extend this work. Furthermore, our findings provide support for theories of knowledge acquisition by demonstrating how personal strengths in knowledge are related to personal strengths in abilities and interests.

## Figures and Tables

**Table 1:** Correlations among demographic variables, abilities, and interests.

No.	Variable	Mean	SD	1	2	3	4	5	6	7	8	9	10
1	Sex	1.5	.5										
2	SES	97.94	10.14	−0.01									
3	Verbal ability	0	1	0.08	0.43								
4	Math ability	0	1	−0.18	0.40	0.70							
5	Spatial ability	0	1	−0.32	0.33	0.60	0.62						
6	Realistic interest	0	1	−0.64	−0.12	−0.16	0.03	0.21					
7	Investigative interest	0	1	−0.29	0.17	0.24	0.37	0.31	0.29				
8	Artistic interest	0	1	0.29	0.08	0.16	0.06	−0.04	−0.04	0.27			
9	Enterprising interest	0	1	−0.18	0.07	0.09	0.13	0.06	0.28	0.30	0.30		
10	Social interest	0	1	0.43	0.07	0.15	0.07	−0.12	−0.19	0.11	0.44	0.30	
11	Conventional interest	0	1	0.36	−0.17	−0.11	−0.19	−0.22	−0.05	−0.10	0.15	0.34	0.32

*Note.* All correlations above 0.01 are significant at *p* < .01. Sex coded as female = 1 and male = 2.

**Table 2:** Latent profile analysis model fit statistics.

Class Model	K	LL	#fp	AIC	BIC	SABIC	Entropy	BLRT	Adj. LMR
2 profiles	2	−3,887,070.924	28	7,774,198	7,774,497	7,774,408	0.827	<0.0001	<0.0001
3 profiles	3	−3,823,429.120	38	7,646,934	7,647,340	7,647,219	0.738	<0.0001	<0.0001
4 profiles	4	−3,768,891.618	48	7,537,879	7,538,392	7,538,239	0.77	<0.0001	<0.3333
5 profiles	5	−3,722,072.520	58	7,444,261	7,444,880	7,444,696	0.779	<0.0001	<0.3333
6 profiles	6	−3,694,846.844	68	7,389,830	7,390,555	7,390,339	0.776	<0.0001	<0.0000
7 profiles	7	−3,672,633.843	78	7,345,424	7,346,256	7,346,008	0.781	<0.0001	<0.0001
8 profiles	8	−3,651,034.688	88	7,302,245	7,303,185	7,302,905	0.791	<0.0001	<0.0001
9 profiles	9	−3,633,432.856	98	7,267,062	7,268,108	7,267,796	0.785	<0.0001	<0.0001
10 profiles	10	−3,622,207.511	108	7,244,631	7,245,440	7,245,440	0.782	<0.0001	<0.0001

*Note.K* indicates the number of profiles in each model. *LL* = log likelihood; *#fp* = number of free parameters; AIC = Akaike information criterion; BIC = Bayesian information criterion; SABIC = sample size-adjusted BIC; BLRT = bootstrapped likelihood ratio test; Adj. LMR = adjusted Lo–Mendell–Rubin test.

**Table 3:** Equality test of gender probabilities across the eight profiles.

	Conventional/Low-Ability	Ambivalent/Low-Ability	Conventional/Average-Ability	Intellectual/Mathematical	Scientific	Realistic/Spatial	Disinterested/Average-Ability	Cultural
Male	0.003	0.992	0.017	0.964	0.996	1.000	0.025	0.109
Female	0.997	0.008	0.983	0.036	0.004	0.000	0.975	0.891

**Table 4:** Equality test of socioeconomic status means across the eight profiles.

	Conventional/Low-Ability	Ambivalent/Low-Ability	Conventional/Average-Ability	Intellectual/Mathematical	Scientific	Realistic/Spatial	Disinterested/Average-Ability	Cultural
Socioeconomic status	−0.580	−0.775	−0.050	0.784	0.151	−0.366	0.281	0.668

**Table 5:** Standardized Beta Coefficients from Regressing Knowledge Domains on Profiles.

	Business	Law	Social Studies	Art	Bible	Literature	Music	Theater	Aeronautics	Electronics	Mechanical	Biology	Math	Physics
Ambivalent/Low-Ability	−0.30	−0.28	−0.37	−0.35	−0.26	−0.35	−0.31	−0.33	−0.20	−0.21	−0.23	−0.28	−0.29	−0.29
Disinterested/Average-Ability	0.07	0.07	0.12	0.14	0.10	0.11	0.12	0.12	0.04	0.00	0.02	0.10	0.03	0.07
Conventional/Low-Ability	−0.31	−0.33	−0.43	−0.33	−0.28	−0.36	−0.30	−0.27	−0.30	−0.31	−0.37	−0.36	−0.38	−0.39
Conventional/Average-Ability	0.01	−0.09	−0.09	0.02	−0.01	−0.05	0.02	0.06	−0.26	−0.27	−0.28	−0.15	−0.27	−0.24
Realistic/Spatial	−0.19	−0.14	−0.18	−0.22	−0.22	−0.24	−0.27	−0.27	0.00	0.05	0.17	−0.10	−0.24	−0.11
Scientific	0.11	0.18	0.23	0.11	0.08	0.11	0.04	0.03	0.30	0.33	0.38	0.22	0.17	0.26
Cultural	0.28	0.23	0.33	0.34	0.30	0.38	0.39	0.37	0.04	0.00	0.00	0.24	0.36	0.24
Intellectual/Mathematical	0.35	0.38	0.43	0.32	0.30	0.43	0.35	0.30	0.44	0.47	0.37	0.39	0.69	0.52

*Note.* Values are standardized beta coefficients. All coefficients greater than .01 are significant at *p* < 0.01. Standard errors ranged from 0.001 to 0.00.

## Data Availability

The Project Talent dataset is protected by a Restricted Use of Data Agreement, which prevents us from making it publicly available. However, we have provided the code used to analyze the data, which is available at our Open Science Framework page.

## Note

1. There are three common definitions for the term “person-centered”, which are related to different methods. Our usage of this term references the clustering of persons, which is related to clustering methods (Woo et al. 2018).

## Supplementary Materials

The following supporting information can be downloaded at https://www.mdpi.com/article/10.3390/jintelligence10030043/s1, Table S1: Interest scales from the Project Talent interest inventory; Table S2: Correlation matrix with all variables used in the study; Table S3: Factor loadings from Exploratory Factor Analysis; Table S4: Average latent class assignment probabilities; Table S5: Project Talent ability test names, test type, number of items, and reliabilities for males/females; Table S6: Project Talent knowledge test names, test type, number of items, and reliabilities for males/females. Figure S1: Scree plot for Exploratory Factor Analysis; Figure S2: Horn’s Parallel Analysis for Exploratory Factor Analysis.
