# Supplementary material for: Interest–Ability Profiles: An Integrative Approach to Knowledge Acquisition
Source: J Intell. 2022 Jul 16;10(3):43. doi: 10.3390/jintelligence10030043 (PMC9326565; doi:10.3390/jintelligence10030043)

SUPPLEMENTAL MATERIALS

Table S1.
Interest Scales from Project Talent Interest Inventory

Realistic	Investigative	Artistic	Social	Enterprising	Conventional
Toolmaker	Surgeon	Musician	Social worker	Personnel administrator	Bookkeeper
Automobile Mechanic	Chemist	Reporter	Elementary school teacher	Credit manager	Bank teller
Electrician	Astronomer	Sculptor	Guidance counselor	President of a large company	Office clerk
Electronics technician	Research Scientist	Author of a novel	College professor	Real estate agent	Certified Public Accountant
Bricklayer	Doctor	Interpreter	High school teacher	Office manager	Typist
House Painter	Biologist	Writer	Religious worker	Banker	Accountant or auditor
Machinist	Laboratory technician	Musical composer	School principal	Salesman	Type setter
Carpenter	Physics	Poet	Sociology	Manage a large store	Make out income tax returns
Operate a crane or derrick	Studying	Artist	Teach children	Sell merchandise to stores	Keep accounts

Table S2

Correlations among Study's Variables

No.	Variable	Mean	SD	1	2	3	4	5	6	7	8	9	10	11	12	13
1	Sex	1.5	.5													
2	SES	97.94	10.14	-.01												
3	Verbal ability	0	1	.08	.43											
4	Math ability	0	1	-.18	.40	.70										
5	Spatial ability	0	1	-.32	.33	.60	.62									
6	Realistic interest	0	1	-.64	-.12	-.16	.03	.21								
7	Investigative interest	0	1	-.29	.17	.24	.37	.31	.29							
8	Artistic interest	0	1	.29	.08	.16	.06	-.04	-.04	.27						
9	Enterprising interest	0	1	-.18	.07	.09	.13	.06	.28	.30	.30					
10	Social interest	0	1	.43	.07	.15	.07	-.12	-.19	.11	.44	.30				
11	Conventional interest	0	1	.36	-.17	-.11	-.19	-.22	-.05	-.10	.15	.34	.32			
12	Literature test	12.12	4.64	-.02	.42	.75	.63	.47	-.10	.26	.18	.10	.11	-.20		
13	Music test	6.12	2.98	.10	.44	.67	.54	.40	-.18	.21	.22	.06	.17	-.12	.66	
14	Social Studies test	14.06	5.42	-.18	.39	.76	.66	.54	.01	.30	.06	.13	.04	-.21	.73	.61
15	Math test	8.28	5.17	-.19	.40	.67	.95	.59	.03	.37	.07	.12	.06	-.21	.63	.54
16	Physical Science test	8.16	4.10	-.30	.33	.62	.69	.61	.12	.41	.01	.07	-.05	-.27	.58	.49
17	Biology test	5.80	2.41	-.20	.29	.59	.54	.51	.07	.32	.05	.04	-.02	-.23	.57	.46
18	Aeronautics test	3.65	2.30	-.43	.27	.42	.49	.53	.23	.35	-.07	.07	-.19	-.30	.45	.36
19	Electronics test	7.20	4.03	-.47	.24	.40	.53	.56	.30	.35	-.13	.07	-.20	-.28	.40	.32
20	Mechanical test	9.65	3.97	-.56	.22	.40	.46	.59	.39	.29	-.17	.10	-.25	-.29	.39	.28
21	Art test	6.09	2.60	.04	.39	.69	.50	.44	-.13	.22	.17	.07	.11	-.16	.66	.61
22	Law test	4.50	1.82	-.16	.30	.60	.52	.46	.04	.24	.06	.12	.02	-.18	.57	.47
23	Business test	4.47	1.98	-.01	.32	.63	.50	.41	-.06	.17	.08	.14	.09	-.07	.56	.50
24	Bible/Religion test	6.87	3.27	.03	.24	.59	.45	.36	-.10	.21	.17	.04	.14	-.11	.55	.46
25	Theater/Ballet test	4.13	1.81	.15	.37	.67	.47	.35	-.20	.14	.19	.07	.17	-.09	.62	.60

Note. All correlations above .01 are significant at $p < .01$. Sex coded as Female = 1, Male = 2.

Table S2 (continued).
Correlations among study's variables

No.	Variable	Mean	SD	14	15	16	17	18	19	20	21	22	23	24	25
1	Sex	1.5	.5												
2	SES	97.94	10.14												
3	Verbal ability	0	1												
4	Math ability	0	1												
5	Spatial ability	0	1												
6	Realistic interest	0	1												
7	Investigative interest	0	1												
8	Artistic interest	0	1												
9	Enterprising interest	0	1												
10	Social interest	0	1												
11	Conventional interest	0	1												
12	Literature test	12.12	4.64												
13	Music test	6.12	2.98												
14	Social Studies test	14.06	5.42												
15	Math test	8.28	5.17	.65											
16	Physical Science test	8.16	4.10	.67	.69										
17	Biology test	5.80	2.41	.61	.54	.62									
18	Aeronautics test	3.65	2.30	.51	.49	.60	.49								
19	Electronics test	7.20	4.03	.49	.53	.66	.49	.63							
20	Mechanical test	9.65	3.97	.50	.46	.56	.51	.59	.66						
21	Art test	6.09	2.60	.62	.50	.49	.48	.39	.34	.33					
22	Law test	4.50	1.82	.60	.51	.51	.47	.45	.43	.45	.51				
23	Business test	4.47	1.98	.57	.48	.44	.42	.35	.35	.37	.53	.52			
24	Bible/Religion test	6.87	3.27	.54	.45	.44	.43	.31	.27	.26	.48	.45	.42		
25	Theater/Ballet test	4.13	1.81	.55	.47	.41	.40	.31	.25	.24	.63	.53	.53	.44	

Note. All correlations above .01 are significant at $p < .01$. Sex coded as Female = 1, Male = 2.

Table S3

Factor loadings from Exploratory Factor Analysis

Variable	Factor 1	Factor 2	Factor 3
Math ability	**.76**	.19	-.01
Verbal ability	**.89**	-.19	.09
Spatial ability	**.56**	**.30**	-.17
Realistic interest	-.26	**.74**	.03
Investigative interest	.23	**.51**	.25
Artistic interest	.18	.03	**.59**
Enterprising interest	.02	**.49**	**.63**
Social interest	.17	-.09	**.66**
Conventional interest	-.19	.01	**.48**
Literature knowledge	**.86**	-.07	.03
Music knowledge	**.79**	-.15	.08
Social studies knowledge	**.82**	.08	.03
Math knowledge	**.74**	.20	-.01
Physical science knowledge	**.66**	**.31**	-.14
Biology knowledge	**.63**	.18	-.11
Aeronautics knowledge	**.46**	**.38**	-.24
Electronics knowledge	**.41**	**.48**	-.27
Mechanical knowledge	**.38**	**.50**	**-.30**
Art knowledge	**.79**	-.13	.03
Law Knowledge	**.65**	.09	-.04
Business knowledge	**.68**	-.02	.04
Bible knowledge	**.65**	-.07	.06
Theater knowledge	**.77**	-.22	.09

Note. Loadings > 0.30 are in bold.

Table S4

Average Latent Class Assignment Probabilities

No.	Profile	1	2	3	4	5	6	7	8
1	Conventional/Low-Ability	.849	.025	.067	.000	.000	.032	.027	.000
2	Ambivalent/Low-Ability	.037	.853	.050	.000	.009	.050	.000	.000
3	Conventional/Average-Ability	.051	.027	.815	.000	.018	.011	.045	.033
4	Intellectual/Mathematical	.000	.000	.000	.906	.034	.000	.006	.052
5	Science	.000	.006	.027	.025	.820	.051	.035	.034
6	Realistic/Spatial	.044	.041	.018	.000	.057	.814	.027	.000
7	Disinterested/Average-Ability	.035	.000	.070	.006	.040	.027	.779	.043
8	Cultural	.000	.000	.051	.045	.037	.000	.042	.826

Note. Probabilities are expressed as proportions.

Table S5

Project Talent ability test names, test type, number of items, and reliabilities for males/females

Test Name	Test Type	Items	Reliability
Vocabulary	Verbal ability	21	.71/.71
Spelling	Verbal ability	16	.60/.56
Capitalization	Verbal ability	33	.85/.83
Punctuation	Verbal ability	27	.72/.73
English usage	Verbal ability	25	.56/.49
Effective expression	Verbal ability	12	.63/.52
Reading comprehension	Verbal ability	48	.86/.84
Mathematics	Quantitative ability	23	.77/.72
Arithmetic reasoning	Quantitative ability	16	.73/.71
Introductory mathematics	Quantitative ability	24	.78/.73
Advanced mathematics	Quantitative ability	14	.52/.42
Visualization in 2D	Spatial ability	24	.81/.80
Visualization in 3D	Spatial ability	16	.70/.59
Mechanical reasoning	Spatial ability	20	.76/.64
Abstract reasoning	Spatial ability	15	.66/.65

Note. Reliabilities are taken from Flanagan et al. (Flanagan, 1964) and are based on the Kuder-Richards Formula 21 otherwise noted. These reliabilities were calculated by averaging the reliabilities across the 9th – 12th grade levels.

Table S6

Project Talent knowledge test names, test type, number of items, and reliabilities for males/females

Test Name	Knowledge Domain	Items	Reliability
Business	Civics	10	.51/.51[a]
Law	Civics	9	.51/.43[a]
Social Studies	Civics	24	.83/.79
Art	Humanities	12	.64/.65[a]
Bible	Humanities	15	.74/.73[a]
Literature	Humanities	24	.72/.70
Music	Humanities	13	.67/.67
Theater	Humanities	8	.52/.59[a]
Aeronautics	Mechanical	10	.63/.34
Electronics	Mechanical	20	.76/.43
Mechanical	Mechanical	19	.66/.48
Biology	Science	11	.57/.51
Math	Science	23	.81/.78
Physics	Science	18	.77/.72

Note. Reliabilities are taken from Flanagan et al. (1964) and are based on the Kuder-Richards Formula 21 unless otherwise noted. These reliabilities were calculated by averaging the reliabilities across the 9th – 12th grade levels.

[a] Estimate based on Kuder-Richards Formula 20

Figure S1

Scree plot for Exploratory Factor Analysis

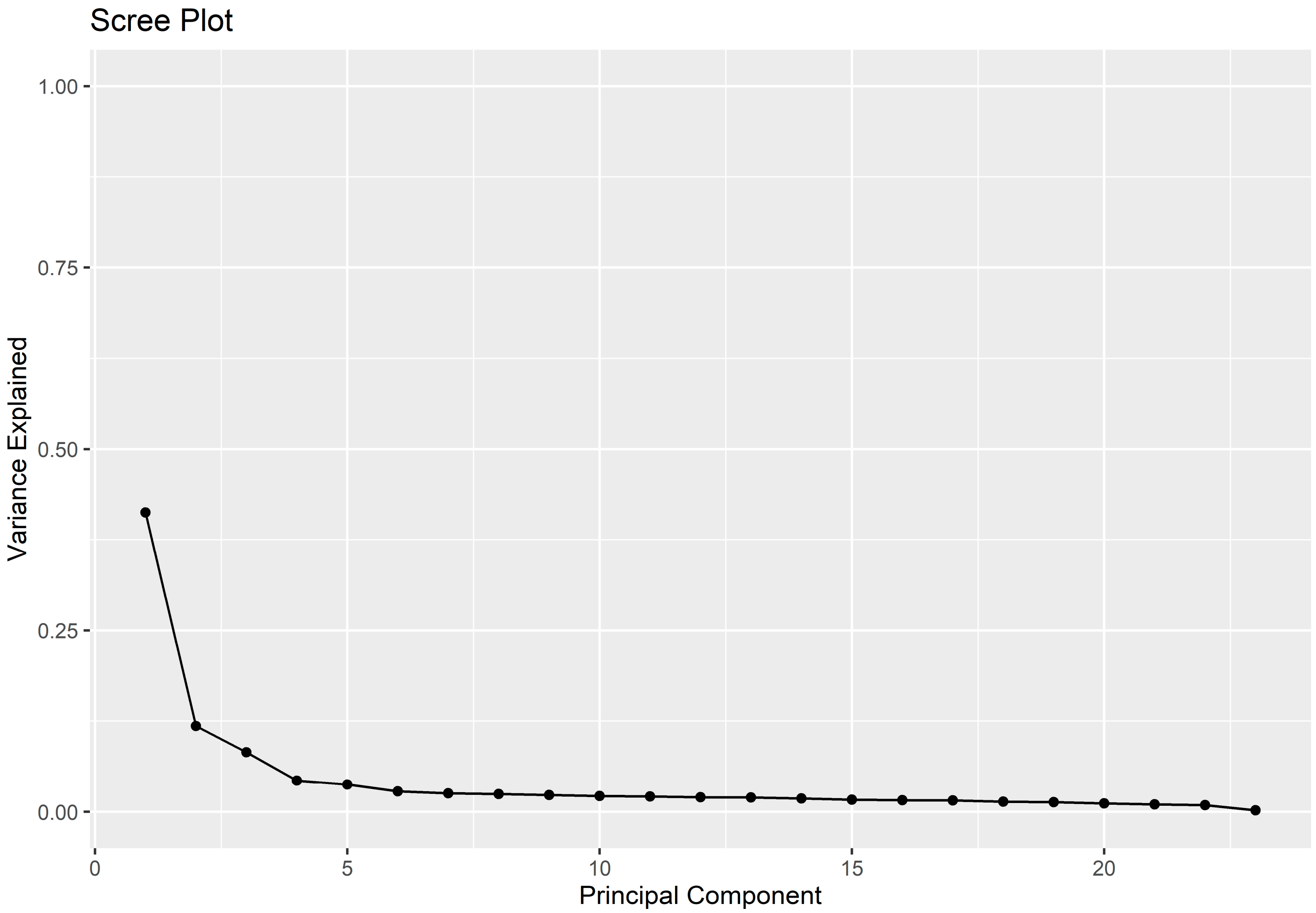

Figure S2

Horn's Parallel Analysis for EFA

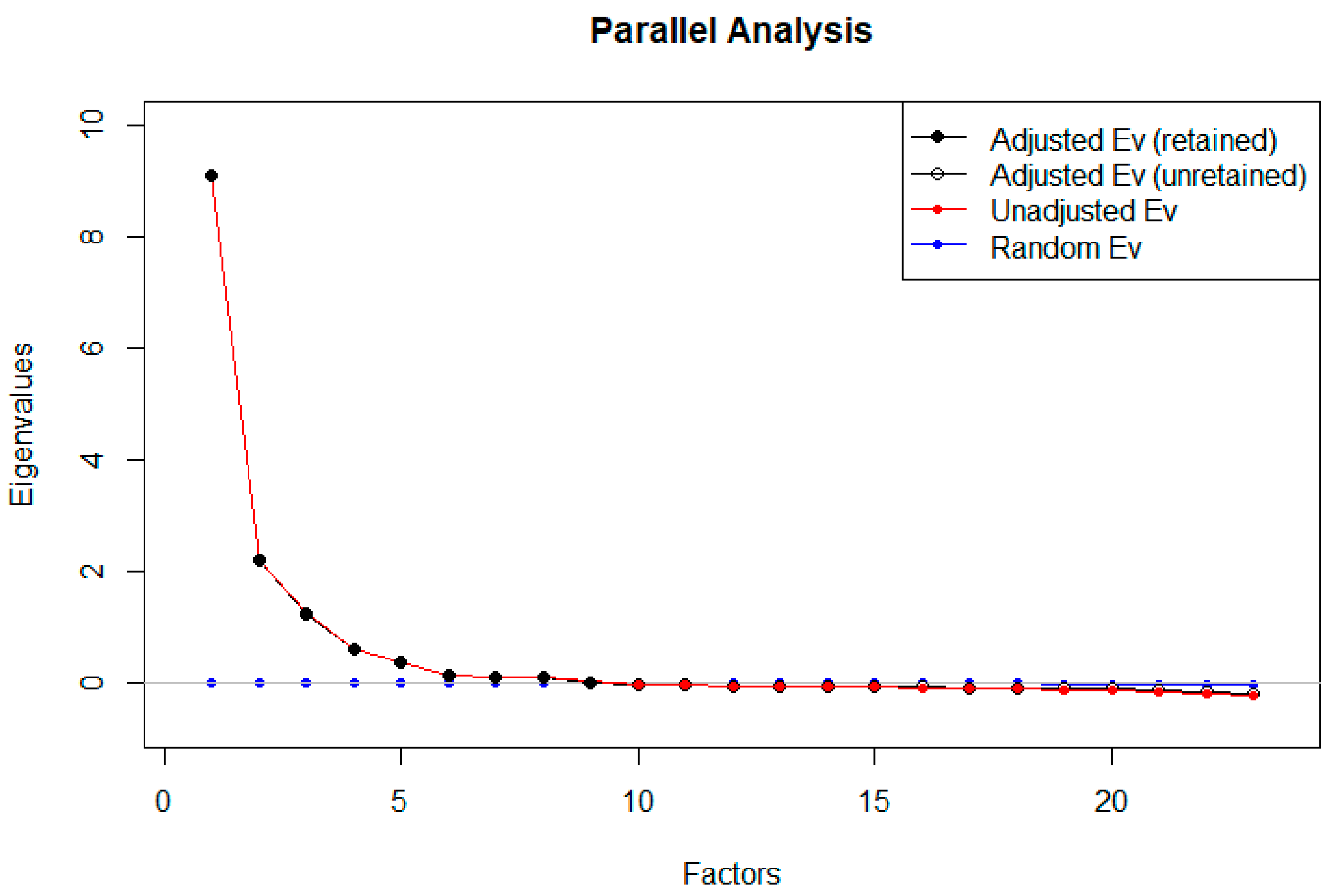

Supplement: Supplementary file 1 [file jintelligence-10-00043-s001.zip › jintelligence-1742795-supplementary.pdf]
